# Dynamic control of asymmetric electromagnetic wave transmission by active chiral metamaterial

**DOI:** 10.1038/srep42802

**Published:** 2017-02-16

**Authors:** Ke Chen, Yijun Feng, Li Cui, Junming Zhao, Tian Jiang, Bo Zhu

**Affiliations:** 1 Department of Electronic Engineering, School of Electronic Science and Engineering, Nanjing University, Nanjing, 210093, China

## Abstract

The asymmetric transmission of electromagnetic (EM) wave can be fully manipulated by chiral metamaterials, but little can achieve real-time and high efficient tunability due to challenges in practically deployable solutions. Here, we proposed a new scheme for flexibly and dynamically controlling the asymmetric EM wave transmission at microwave frequencies using planar metamaterial of deep subwavelength thickness incorporated with active components of PIN diodes. The asymmetric transmission of linearly polarized EM wave exhibits a high efficiency and a pronounced real-time continuous tunability controlled by the external stimulation of voltage biasing. In addition, the asymmetric transmission effect can be well preserved at large oblique incident angle up to ±70°. The design principle and EM performance are validated by both full wave simulations and experimental measurements. Such dynamically controllable chiral metamaterial may provide robust and flexible approach to manipulate EM wave propagation, as well as to facilitate EM device integration to create diverse functionalities.

From bio-molecules, polymers to crystals, chiral molecules and structures are ubiquitously in the real physical world, but the chiral effect in these naturally occurring media is quite weak. Metamaterials, the artificial composites with subwavelength structural inclusions (or termed as meta-atoms), have been emerged as a promising platform to realize custom-designed chiral response which is much stronger than that of natural materials^1^,^2^,^3^. By breaking the mirror symmetry, these chiral metamaterials can be utilized to achieve many abnormal phenomena including negative refraction^2^,^4^,^5^ and handedness-dependent non-linear response^6^, as well as other functional devices aiming to manipulate the polarization states of electromagnetic (EM) waves with novel features like asymmetric transmission^7^,^8^,^9^,^10^, giant optical activity^4^,^11^ and circular dichroism^3^,^12^, etc. Particularly, the asymmetric EM wave transmission is a fascinating physical phenomenon, which is recognized as a difference in total transmission energy between forward and backward propagations either for circularly or linearly polarized EM waves. However, conventional devices to achieve asymmetric transmission with non-reciprocity methods are usually heavy, complex and bulky, which could not be readily integrated into modern compact systems^13^. The alternative approaches in reciprocal and planar fashions by chiral metamaterials enable asymmetric transmission with unique advantages of passive operation, light-weight and especially subwavelength thickness, which have already manifested their potential uses in many real-world applications, such as spectroscopy, signal processing and communications.

Although metamaterials can be designed in various forms, the vast majority of them are composed of passive meta-atoms, such as structured metallic or dielectric inclusions with fixed EM wave performances, which cannot be reconfigured in real-time once fabricated, limiting their capability in manipulating the EM wave propagation. Therefore, substantial efforts are now focused on metamaterials with switchable, tunable or reconfigurable properties actuated by thermal^14^, electric bias^15^,^16^,^17^ and mechanical deformation^18^,^19^, and many meta-devices including switchable absorbers^16^,^20^, ultrafast EM wave modulators^15^, reconfigurable lenses^21^, as well as devices with tunable optical activity or polarization^22^,^23^,^24^,^25^,^26^,^27^ have been widely implemented in various applications, thus making metamaterials controllable with external stimulations realistic schemes. Remarkably, in the microwave regime, PIN diodes has been demonstrated as a well-controllable active component that could be hybridized with the meta-atoms to dynamically manipulate the EM responses, which is more preferable than other alternative routes like thermal or structural deformation to realize switchable or tunable functional devices (especially when requiring fast response time), such as tunable frequency selective surfaces (FSS)^28^,^29^,^30^, absorbers^16^,^20^, polarizers^27^ and reconfigurable antennas^31^,^32^,^33^.

Although asymmetric transmission of EM wave is successfully realized by various types of metamaterials with symmetry-broken chiral configurations, its performance is usually fixed which lacks of dynamic and reconfigurable control, and inherently limits its practical uses^7^,^8^,^9^,^10^,^34^,^35^,^36^,^37^,^38^,^39^. Recently some theoretical proposals have been reported to realize dynamic asymmetric transmission for certain polarized EM wave^23^,^25^, but practical challenges are still needed to be tackled for real-time tunability with practically deployable solutions. Here in this paper, we proposed a new strategy to realize dynamic control of the asymmetric transmission for certain linearly polarized EM waves at microwave frequencies. Through elaborately designing a chiral meta-atom incorporating active component of PIN diode, without much extra complexity in the structural design and optimization, we are able to dynamically control over the transmission property. Benefit from the transient state of the PIN diode as the bias voltage gradually changes, the asymmetric transmission can be continuously and flexibly manipulated at will in real-time according to the external voltage stimulation. In addition, the asymmetric transmission effect can be well preserved at large oblique incident angle up to ±70° for both wave polarizations. The dynamic performances have been validated both from the simulation and measurement on fabricated prototype with good coincidence with each other. We expect such dynamically controllable chiral metamaterial could provide new ways for active and reconfigurable functionality, which may be applicable to versatile compact EM device designs.

## Results

### Concept and experimental verification

**Figure 1:**
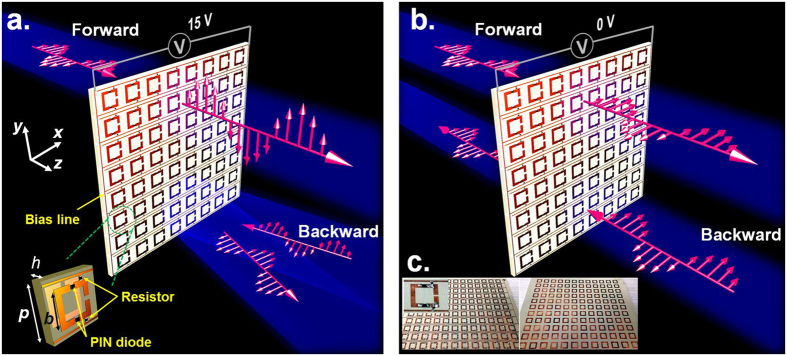
Active chiral metamaterial for dynamic control of the asymmetric EM wave transmission. (**a**) Biased by an external DC voltage source of 15 V, the chiral metamaterial can transform the *x*-polarized EM wave along forward direction (+*z*) into *y*-polarization with high transmittance, while reflect the opposite incoming EM wave to its specular direction, leading to an asymmetric EM wave transmission. The unit cell is shown on the lower-left part with optimized geometric parameters of *p* = 12 mm, *h* = 1.52 mm, *b* = 8.2 mm. The wire width of the square SRR structure and bias line are 1.3 mm and 0.4 mm, respectively. The width of the slots on both sides is 0.6 mm. The distance between the bias line and the SRR structure is 0.9 mm. (**b**) Biased by an external DC voltage source of 0 V, the *x*-polarized EM wave either propagating along forward or backward direction can pass through the chiral metamaterial without much attenuation, resulting in symmetric EM wave transmission. (**c**) The photograph of the frontside (left) and the backside (right) of the fabricated sample. Inset shows the enlarged view of the unit cell.

The most straightforward approach to generate strong chiral response is to mimic the construction of helical molecule in nature by suitably scaling the overall dimension and thus synthesizing real three-dimensional (3D) chiral meta-atoms that look like helices^3^,^40^,^41^,^42^,^43^,^44^. Such method can be readily reduced to their planar counterpart as recently demonstrated, where the total thickness of the meta-atoms along the wave propagation direction is usually much smaller than the operational wavelength^7^,^10^,^12^,^45^,^46^,^47^,^48^. By placing two metallic square split ring resonators (SRRs) twisted 90° to each other on two sides of the dielectric substrate, we both theoretical and experimentally demonstrate that high asymmetric transmission of linearly polarized EM wave can be successfully realized^10^. Here, the proposed active chiral metamaterial is based on such twisted SRRs structure, and the configuration of the unit cell is sketched in the lower-left part of Fig. 1. In order to achieve a tunable metamaterial, two metallic arms of the front SRR structure are cut and then connected by two identical PIN diodes working with same states to dynamically control the structural resonances. The two SRRs are shaped with identical geometric parameters and separated by a dielectric layer of Taconic RF-35A2 with thickness of 1.52 mm (with a relative permittivity of 3.5 and a loss tangent of 0.0018), but the one on back side has no PIN diodes and is twisted by an angle of 90° in the *x-y* plane with respect to the front one. The bias lines are etched on the front side to connect the active lumped elements to the external DC voltage sources. Two chip resistors (20 K ohm) serving as the currents choke are embedded between the metallic bias lines and resonant structure to get rid of unwanted coupling between these adjacent metallic parts that may further spoil the tunable performance, as well as the high transmittance. All the metallic films used in the design are made of copper with a thickness of 0.018 mm.

Asymmetric transmission of linearly polarized EM wave is usually associated with the efficient cross-polarization conversion when the incident EM wave impinging on a certain side of the chiral metamaterial, while such conversion is quite different for the same polarization incident along the opposite direction. For a linearly polarized wave propagating along the forward direction (+*z*) that normally impinges on the structure, the incident and transmitted electric fields can be decomposed into two linearly polarized *x*- and *y*-components^8^,^10^,^44^:


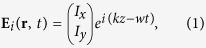



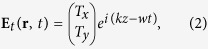


where * ω* is the frequency, *k* is the wave vector, and *I*_*x*_, *I*_*y*_ and *T*_*x*_, *T*_*y*_ represent the complex amplitudes of the incident and transmitted wave, respectively. Transmission Jones matrix can be applied to better intuitionally relates the incident field to the transmitted field with consideration of the polarization states:


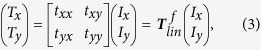


where the superscript ‘*f*’ indicates the forward propagation, and the subscript ‘*lin*’ indicates the linear polarization. Since there does not involve non-reciprocal component in the medium, the reciprocal theorem can be applied to derive the transmission matrix for the EM wave propagating along backward direction (−*z*), which gives (the superscript ‘*b*’ indicates the backward propagation):


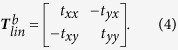


Tunable metamaterials can change the functionalities or their performances upon the external stimulations, which is the most advantage over conventional metamaterials. Here, we start the proposal with PIN diodes working at two extreme states, namely, the ON or OFF state, to show that the dynamically switchable asymmetric transmission can be realized by the active chiral metamaterials around the working frequency of 9.2 GHz. In the present scheme, the PIN diodes are selected from the off-the-shelf commercial products whose overall dimensions are negligible compared to the operational wavelength, thus minimizing the parasitic effect and their mutual coupling with metallic cut wires. This PIN diode can be molded as an inductance of 0.7 nH serially connected with a resistance of 2 ohm at the ON state, while a capacitance of 0.15 pF at the OFF state. One can flexibly alter the working state of the PIN diode from an insulator to an almost conductive one by choosing appropriate bias voltage. The performances of the proposed chiral metamaterial has been first optimized in full-wave simulations through a commercial software. Then, a planar sample composed of an array of 12 × 12 unit cells with the bias lines addressing every unit cell is implemented and fabricated through standard printed circuit board (PCB) technique. The prototype of the sample is shown in Fig. 1(c). The active chiral metamaterial is experimentally characterized after the fabrication and details of the measurement can be found in Methods section.

**Figure 2:**
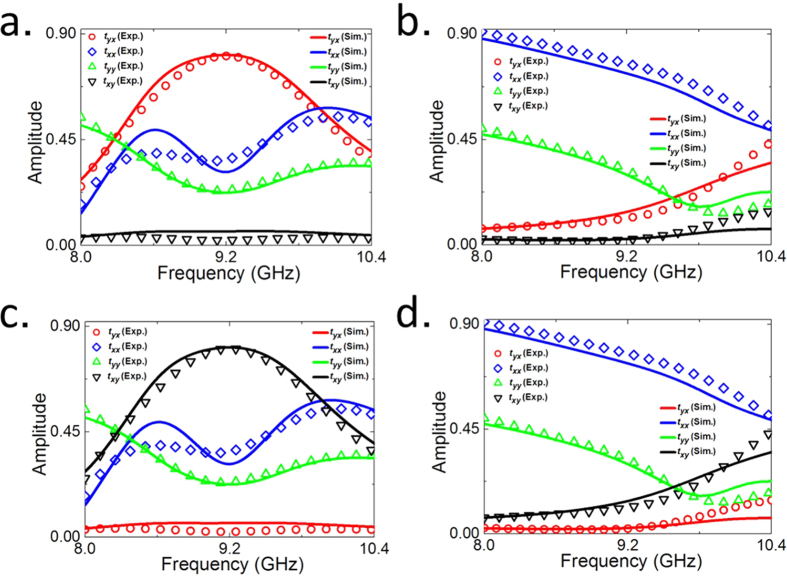
Simulated and measured switchable properties of the active chiral metamaterial. Forward transmission Jones matrix with the PIN diodes in the metamaterial (**a**) switched on, or (**b**) switched off. Backward transmission Jones matrix with the PIN diodes in the metamaterial (**c**) switched on, or (**d**) switched off.

**Figure 3:**
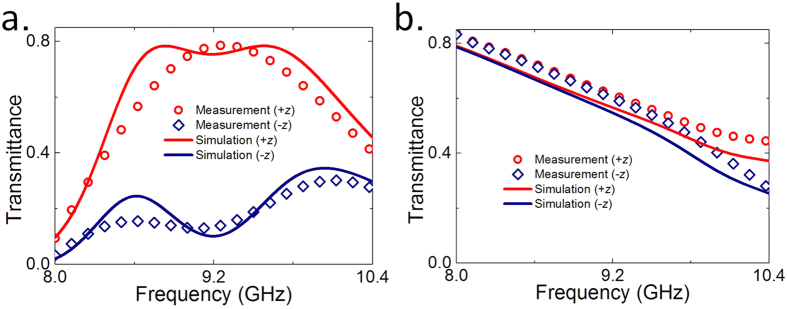
Simulated and measured results of forward and backward total transmittance under the illumination of *x*-polarized plane wave. The PIN diodes are (**a**) switched on by bias voltages of 15 V, or (**b**) switched off by bias voltages of 0 V.

The switchable performances of the proposed active chiral metamaterials are schematically illustrated in Fig. 1, and the corresponding simulated and measured results of the transmission Jones matrix for plane wave along forward and backward direction under the bias voltage of 15 V or 0 V are shown in Fig. 2, respectively. Commercial software Ansys HFSS is used to perform the simulations. When biased by the external DC voltage source of 15 V that exceeds the threshold voltage of diodes, the PIN diodes are switched on to act as conductive parts in the SRR structures. In this case, the overall chiral metamaterial structure is similar to that of ref. 10, which can provide high transmission of *x*-polarized EM wave along forward direction by converting the incidence into *y*-polarization, but blocks the *x*-polarized EM wave that incidents from the opposite side of the metamaterial. As illustrated in Fig. 2(a), the forward cross-polarization transmission coefficient *t*_*yx*_ experiences a transmission peak with a maximum reaching 0.82 at the operating frequency of 9.2 GHz both in simulation and measurement, while the co-polarization transmission coefficient *t*_*xx*_ nearly reduces to its minimum of less than 0.3. The backward co-polarization transmission coefficient *t*_*xx*_ is same as that of forward propagation due to the reciprocity principle, but the *t*_*yx*_ remains close to zero, as shown in Fig. 2(c). The remarkable difference of *t*_*yx*_ between two EM waves with opposite propagation directions will results in a high asymmetric cross-polarization-conversion for *x*-polarized EM wave, thereby, leading to the total transmitted energy being quite different. As Fig. 3(a) shows, the total transmittance (|*t*_*yx*_|^2^ + *t*_*xx*_|^2^) of backward propagation is only about 0.1 at the resonant frequency, indicating 10% of the total incident energy can efficiently transmit through the metamaterial. However, the transmittance is dramatically improved and reaches to about 0.8 for the forward propagation. In addition, Fig. 2(a) and (c) indicates that the cross-polarization transmission coefficient of *t*_*yx*_ and *t*_*xy*_ will interchange with each other once the propagation direction is reversed due to the reciprocity theorem, which exactly complies with the theoretical prediction in Equations (3 and 4). All these results show good agreements between full-wave simulations and measurements, therefore validate that high asymmetric transmission can be obtained through voltage biasing on the proposed active chiral metamaterial.

On the contrary, there is weak asymmetric transmission occurs for the case of PIN diodes without voltage biasing. When the PIN diode is switched off, it works simply as a capacitance that destroys the SRR structure and further the chiral response of the metamaterial, leading to direct transmission without polarization conversion for *x*-polarized EM wave coming from either side of the metamaterial. This is demonstrated in Fig. 2(b) and (d), where a high co-polarization transmission coefficient (*t*_*xx*_) of about 0.8 is observed for both forward and backward propagated EM waves, while the cross-polarization conversion is quite weak and the corresponding transmission coefficient is reduced to a minimum close to zero. The total transmitted energy is shown in Fig. 3(b), where the transmittance of *x*-polarized EM wave along forward direction is nearly the same as that of *x*-polarized EM wave along backward direction, therefore, demonstrates that weak asymmetric transmission (or highly symmetric transmission) can be obtained by switching off the PIN diodes of the active chiral metamaterial.

**Figure 4:**
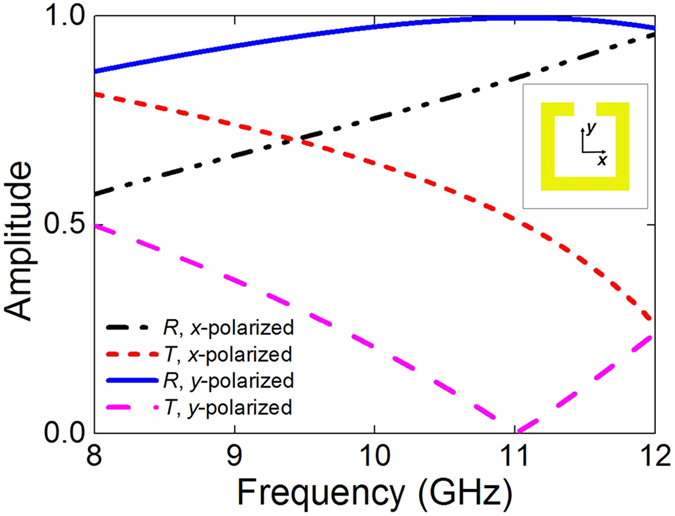
The spectrum responses of a single metallic SRR under the normal illumination of a plane wave incidence.

As mentioned above, the asymmetric transmission is actually attributed to the cross polarization transmission components, which are different for certain polarized EM wave between forward and backward transmission. Achieving high cross-polarization-conversion for linearly polarized EM wave requires anisotropic electric response and magneto-electric mutual coupling in the structure. Anisotropic electric property can be easily design by ultrathin patterned metallic sheets, since the frequency-selective-surface theory provides extensive literature to generated arbitrary electric response^49^. However, such single-layered structures cannot offer magneto-electric coupling, the most important condition necessary for high cross-polarization conversion. Indeed, the fundamental limit of conversion efficiency is only 25% for a single layer^50^. Therefore, we adopt two-layered metallic SRR structure in this paper, but the thickness is only 0.046*λ (λ* is wavelength in free space), still much smaller compared to the wavelength. A metallic SRR structure could have polarization-dependent responses to the incident EM wave^51^, and the spectrum responses of the single metallic SRR structure in this paper (the one on backside of the unit cell) is shown in Fig. 4. For an *x*-polarized incidence, the wave energy can propagate through the structure with a high transmission due to the existing slot along *x*-direction. On the contrary, the *y*-polarized incidence is near totally reflected when impinging onto the structure. The single SRR structure allows the EM wave to pass through it only if the incident polarization is in parallel to the slot. In our design, when all the PIN diodes are switched on, the EM responses of the unit cell is similar to that of two SRR structures twisted by an angle of 90° to each other on two sides of a dielectric spacer. Therefore, a forward *x*-polarized propagating wave penetrates through the first SRR but interacts with the second one, which in turn interacts with the first one due to the near-field magnetic coupling between the adjacent SRRs and finally a linear *y*-polarized EM wave is radiated. For the backward case, an *x*-polarized EM wave first hits the second SRR, which can hardly be coupled into the structure and mostly got bounced to the specular direction with respect to the incidence, resulting in a low co-polarization transmission. However, once the PIN diodes are switched off, the second SRR will have slots on both *x*- and *y*-directions. Thus the highly polarization-dependent property of the second SRR structure is destroyed, leading to high co-polarization symmetric transmission for *x*-polarized incidences from both sides of the metamaterial.

**Figure 5:**
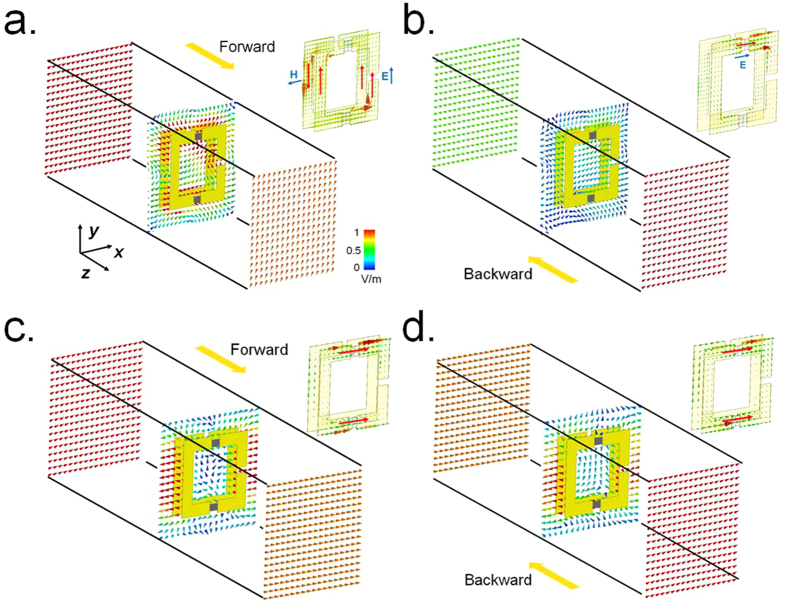
Electric field and surface current distributions. Normalized electric field distributions at 9.2 GHz for an *x*-polarized wave incident along the (**a**) forward and (**b**) backward direction when the diodes are switched on, and results for the an *x*-polarized wave incident along (**c**) forward and (**b**) backward direction when the diodes are switched off. The three slices are recorded at 10 mm from the sample front surface, the middle of the dielectric spacer and 10 mm from the sample back surface, respectively. The upper-right panel of each figure shows the corresponding surface current distribution on the metallic SRR structures at 9.2 GHz.

Electric field evolution and the surface current distribution of the SRR structure are shown in Fig. 5 to further elucidate the working mechanism of the active chiral metamaterial. When the diodes are switched on and a forward *x*-polarized incidence impinges on the structure, currents will be induced in the metallic SRR and results in antiparallel currents on one lateral side of the two resonators attributed to their conjugated arrangement, while parallel currents on the other lateral side, as shown in the upper-right panel of Fig. 5(a). Therefore, a magnetic dipolar resonance along *x*-direction and an electric dipolar resonance along *y*-direction can be excited. The coupled electric and magnetic fields finally lead to a polarization rotation at the resonant frequency, namely a *y*-polarized transmitted EM wave, as demonstrated by the corresponding electric field evolution in Fig. 5(a). However, the resonant feature of the unit cell is quite different when the incidence is inverted to the backward direction. As illustrated in Fig. 5(b), the incidence first hits the second SRR and its magnetic resonance cannot be excited, which therefore results in a comparably weak coupling and a low level of backward co-polarization transmission. Such excitation of different modes for forward and backward cases successfully gives rise to a comparable high asymmetric transmission for *x*-polarized incidence. On the contrary, once the PIN diodes are switched off, the resonant features of the unit cell are similar between forward and backward propagating wave, resulting in high co-polarization symmetric transmission for *x*-polarized incidences from both sides of the metamaterial, as shown in Fig. 5(c) and (d).

### Continuously tuning of asymmetric transmission

The key to realize tunable asymmetric transmission is to create changeable resonant properties in the unit cell structures. Possible alternative active components could be the varactor diodes whose capacitance can be dynamically tunable but their resistance keeps as a constant value, by which, however, only the resonant frequency but not the strength of resonance could be potentially changed^52^. In this regard, PIN diodes are preferable in cases where the continuously tuning of the resonant strength or the surface impedance is needed, due to their resistance can be tuned over several orders between ON and OFF states at microwave frequencies^20^. As the demonstration in the next, we will show that continuously tuning of the asymmetric transmission can be dynamically controlled by the proposed active metamaterial.

**Figure 6:**
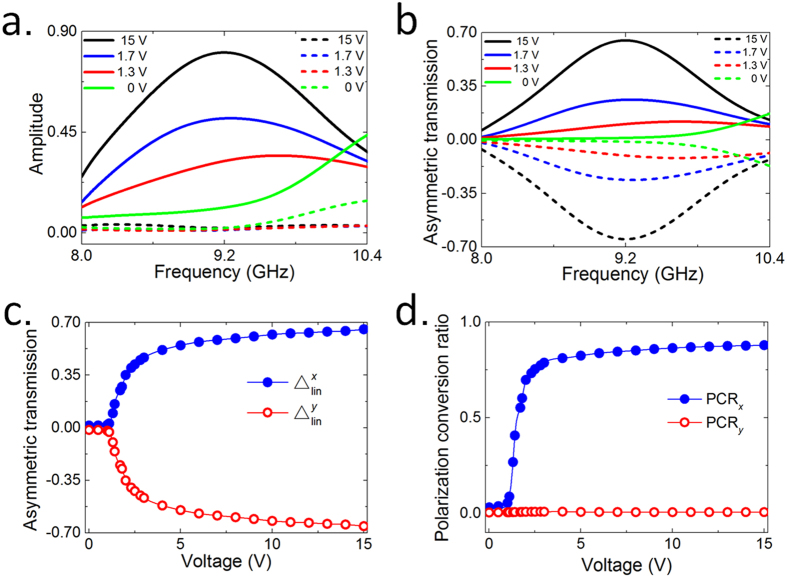
Continuously tuning of asymmetric transmission by proposed active chiral metamaterial. (**a**) The cross-polarization components of *t*_*yx*_ (solid lines) and *t*_*xy*_ (dashed lines) of the forward transmission for *x*-polarized EM wave under different bias voltages, and (**b**) the corresponding results of asymmetric transmission parameter 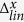. (**c**) Bias-voltage-dependent asymmetric transmission parameters at the target frequency of 9.2 GHz. (**d**) Bias-voltage-dependent polarization conversion ratio at the target frequency of 9.2 GHz.

To quantify the bias-voltage-controlled asymmetric transmission effect, we here introduce asymmetric transmission parameter Δ, which is usually utilized to characterize the asymmetric transmission property and can be defined as the difference between the total transmissions of EM waves along two opposite directions. It can be calculated by


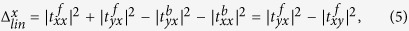



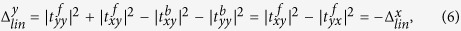


where the subscript “*lin*” indicates the linearly polarization. As illustrated in Fig. 6(a), if we gradually increase the resistance of the PIN-diodes on the front side by reducing the bias voltage, the near-field coupling effect between the two layers will change as well, leading to the cross-polarization transmission coefficient for *x*-polarized EM wave (*t*_*yx*_) along forward direction gradually reduced to zero observed at the working frequency of 9.2 GHz, while the ones for *y*-polarized EM wave (*t*_*xy*_) remains almost unchanged, as it is not strongly affected by the coupling effect but is intrinsically restrained close to zero due to the particular bi-anisotropic responses of the SRR structure on the backside. Hence, the asymmetric transmission parameter 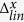 gradually decreases from about 0.67, the maximum across the entire frequency band of 8–10.4 GHz, to zero as the bias voltage reduces to 0 V, as shown in Fig. 6(b). Here we also use the polarization conversion ratio (PCR) to quantify the performance of the cross-polarization conversion for *x*- or *y*-linearly polarized incidence propagating along forward direction. The PCR is defined as the ratio of the cross-polarization transmittance to the total transmittance, which can be calculated by


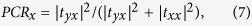



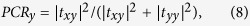


where subscript “*x*” or “*y*” indicates the *x*- or *y*-polarization incidence, respectively. The resulting PCR for both polarizations is illustrated in Fig. 6(d), where the PCR_*x*_ gradually increases and finally reaches to about 0.9 as the voltage changes from 0 to 15 V. Meanwhile, the PCR_*y*_ are negligible, due to the component *t*_*xy*_ always stays close to zero as indicated in Fig. 6(a). The above results suggest that by appropriately choosing the external bias voltage to determine the working state of the PIN diodes from an insulator to the almost conductive one, the cross-polarization transmission for a certain linearly polarized EM wave can be altered at will, further leading to a desirable asymmetric transmission parameter.

### Oblique performances

**Figure 7:**
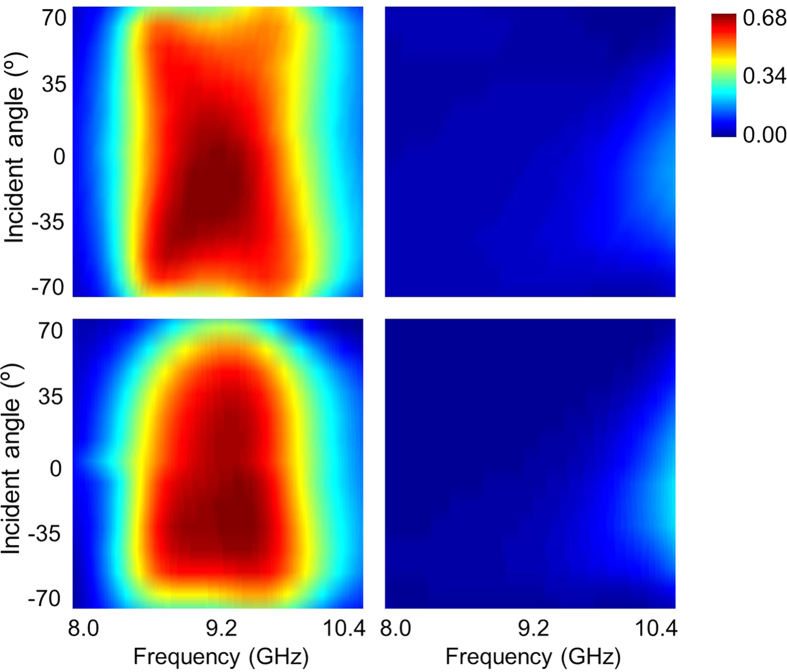
Simulated and measured results of asymmetric transmission parameter 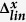
 **for TE-polarized oblique incidence.** Simulated results with PIN diodes (**a**) switched on, or (**b**) switched off, and the corresponding measured results are shown in (**c**,**d**), respectively.

**Figure 8:**
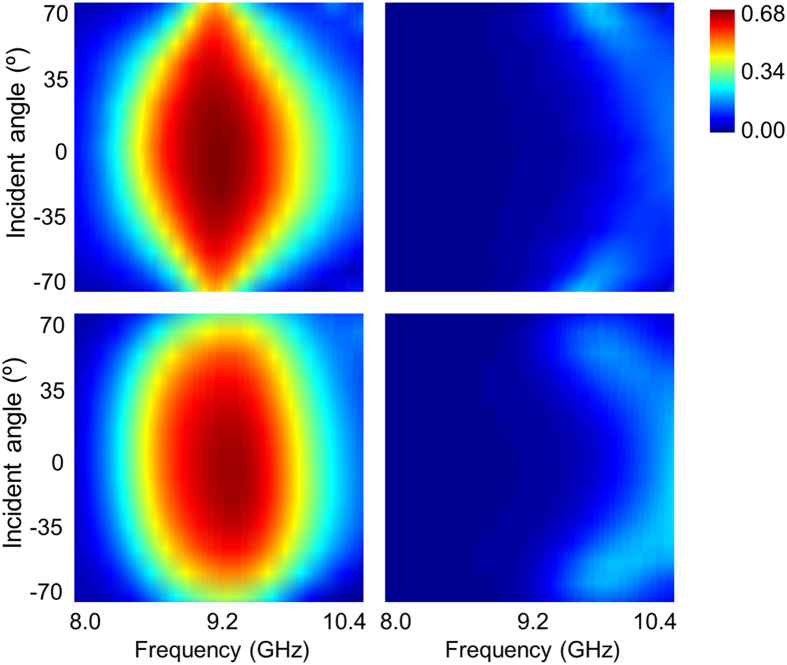
Simulated and measured results of asymmetric transmission parameter 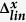
 **for TM-polarized oblique incidence.** Simulated results with PIN diodes (**a**) switched on, or (**b**) switched off, and the corresponding measured results are shown in (**c**,**d**), respectively.

The robust angular-dependent performance is an important criterion to evaluate the metamaterials in many practical applications. However, the chiral responses usually weakens in intrinsically chiral metamaterial under oblique incidence. Hence, we have investigated an acceptable incident angle range where the dynamical control of asymmetric transmission is achievable. For the simplicity and view, we only plot the results of either the PIN diodes are switched off or on. Figure 7(a) and (b) plot the simulated parameter 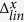 versus different angles of TE wave incidence (the magnetic field parallel to *y-z* plane while the electric field perpendicular to it) with the bias voltage of 15 V and 0 V, respectively. It is clearly that little degradation of the high asymmetric transmission occurs until the incident angle up to 70° when the PIN diodes are in ON state, while the asymmetric transmission remains very weak close to zero across the entire working frequency band when the PIN diodes are switched to OFF state. The results for TM incidence are plotted in Fig. 8, where the robust angular-dependent performances are also observed only except that the high asymmetric transmission seems to degrade rapidly as the variation of frequency at large incident angle (i.e. 70°) when the PIN diodes are switched to ON state. As experimental verifications, the measured results illustrated in Figs 7(c,d) and 8(c,d) are in good coincidence with the simulation predictions when taking the consideration of the fabrication tolerance and imperfect assembly.

## Discussion

We have realized real-time tunability of active chiral metamaterial structure with specific metallic cut wires connected by PIN diodes, which can be changed from an insulator to an almost conductive one upon corresponding electrical biasing. The asymmetric EM wave transmission for certain linearly polarized wave is verified both by full-wave simulations and experiments with good agreements between them. In addition, the performances of high asymmetric transmission are robust to oblique incident angles up to about 70° both under TE and TM polarized incidences. It is worth mentioning that the switchable properties are electrically controlled by the bias voltages, where the switchable speed could reach as fast as a few microseconds depending on the dynamic behavior of the PIN diodes employed in the metamaterial, thus offering robust and flexible approaches to dynamically manipulate electromagnetic wave propagation. The active chiral metamaterials can be also scaled to other frequency bands such as terahertz or optical regime, where the active components may be realized by graphene or semiconductor materials^15^,^17^. In general, we believe that the design principle is not limited to asymmetric transmission for linearly polarized EM wave but can also be applied to realize other device functionalities, such as to realize handedness switching, dynamic polarization control and signal modulation.

## Methods

### Experimental setup

The transmission measurements are carried out in a microwave anechoic chamber to minimize the reflections from surroundings as well as to avoid the interference from the environment. A pair of lens antenna serving as the emitter and the detector is connected to the two ports of a vector network analyzer (Agilent N5245A) by coaxial-cables, respectively. A DC voltage source is utilized to give a precise control over the working state of PIN diodes. The polarization state of the incidence can be adjusted by rotating the emitter with an angle of 0° or 90° to obtain either *x*- or *y*-linearly polarized EM wave, and the detector can be reconfigurable to co-polarization or cross-polarization states by rotating with an angle of 0° or 90° with respect to the emitting antenna. To characterize the transmission properties of the active chiral metamaterial, the unity transmission coefficient is calibrated by the direct co-polarization transmission between the two antennas. For normal incidence, the fabricated sample is placed between the two antennas and parallel to the lens aperture. For oblique incidence cases, the sample is tilted to different angles gradually varying from −70° to 70° with respect to the input EM wave mode, while the other components in the experiment setup are kept unchanged.

## Additional Information

**How to cite this article**: Chen, K. *et al*. Dynamic control of asymmetric electromagnetic wave transmission by active chiral metamaterial. *Sci. Rep.* **7**, 42802; doi: 10.1038/srep42802 (2017).

**Publisher's note:** Springer Nature remains neutral with regard to jurisdictional claims in published maps and institutional affiliations.
